# Supplementary material for: Effect of Methionine on Gene Expression in Komagataella phaffii Cells
Source: Microorganisms. 2023 Mar 29;11(4):877. doi: 10.3390/microorganisms11040877 (PMC10143545; doi:10.3390/microorganisms11040877)
Supplement: Supplementary file 1 [file microorganisms-11-00877-s001.zip › Figure S1. Analysis of pPICZ-PHO1 plasmid and PAP1-X33 K. phaffii strain generated in this study.pdf]

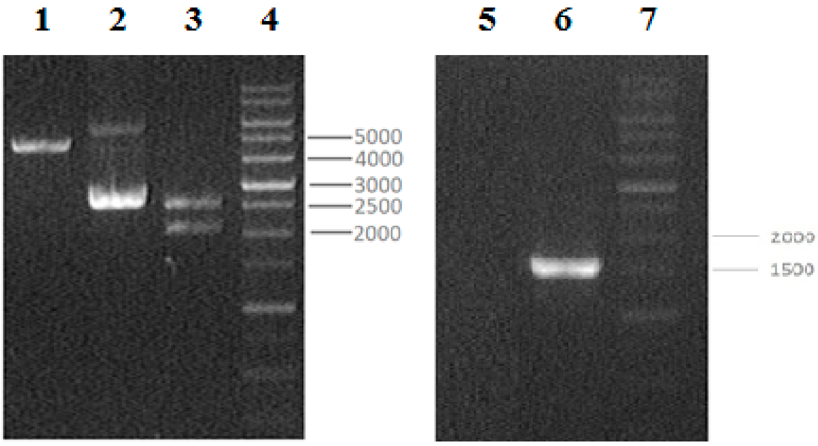

(**a**)

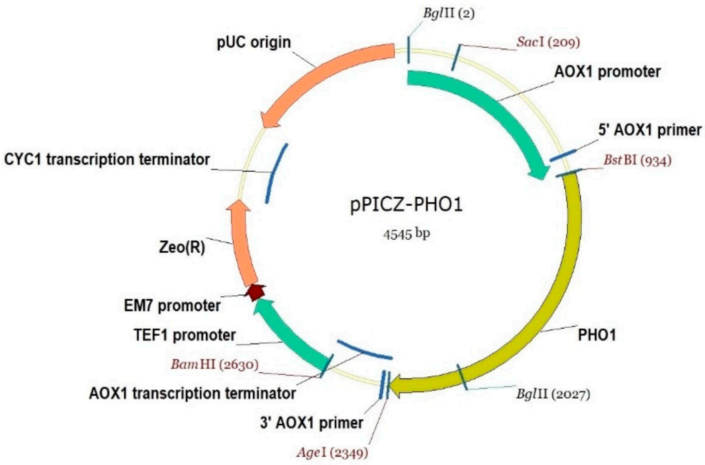

(**b**)

Medium / Strains	Genotype	Md (glucose)	MMet (methanol)
**PAP1-X33**	**PAOX1-PHO1 ZeoR**	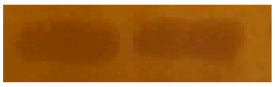	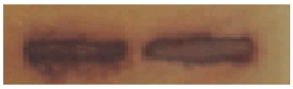
**X-33 original strain**	**Wild type**	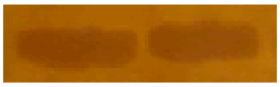	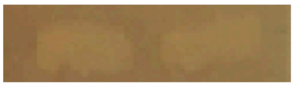

(**c**)

Figure S1. Analysis of pPICZ-PHO1 plasmid and PAP1-X33 *K. phaffii* strain generated in this study. (a) PICZ-PHO1 plasmid was analysed using restriction and PCR. Electropherogram demonstrates: results of restriction analysis: lane 1—pPICZ-PHO1 plasmid, cut at the BamHI site, fragment size ~ 4535 bp, lane 2—pPICZ-PHO1 plasmid, native plasmid, lane 3—pPICZ-PHO1 plasmid, cut at the at the BglII sites, fragment sizes ~ 2520 and 2025 bp, lane 4—1kb DNA ladder (Evrogen, Russia); results of PCR amplification with primer 5`AOX1 (5′-GACTGGTTCCAATTGACAAGC-3′) that is complementary to *AOX1* gene promoter and PHO1-AgeI-R (5′-ATTACTACCGGTTTATGACAAGTCATCCCAGAAG-3′)

that is complementary to *PHO1* reporter gene: lane 5—negative control, no plasmid DNA, lane 6—pPICZ-PHO1 plasmid as a template, fragment size ~ 1505 bp, lane 7—1kb DNA ladder (Evrogen, Russia). (b) Map of pPICZ-PHO1 plasmid, containing *PHO1* acid phosphatase coding sequence under control of the *AOX1* gene promoter. *ZeoR* corresponds to zeocin resistance gene under the control of the yeast *TEF1* promoter and the EM7 bacterial promoter. CYC1 – transcriptional terminator. pUC – bacterial replication origin. (c) Results of qualitative analysis of reporter acid phosphatase (ACP) activity on the surface of *K. phaffii* PAP1-X33 and X-33 strain colonies grown on media with different carbon sources. PAP1-X33 strain carries a *PHO1* reporter gene under the control of the *AOX1* gene promoter. Thus, on when grown on media with methanol this strain demonstrates ACP activity due to induction of *AOX1* promoter. On media with glucose *AOX1* promoter is repressed and ACP is not synthetized.
