# Supplementary material for: Effect of Methionine on Gene Expression in Komagataella phaffii Cells
Source: Microorganisms. 2023 Mar 29;11(4):877. doi: 10.3390/microorganisms11040877 (PMC10143545; doi:10.3390/microorganisms11040877)
Supplement: Supplementary file 1 [file microorganisms-11-00877-s001.zip › Table S2. Number of reads for each sample analyzed by RNA-sequencing.pdf]

Table S2. *Number of reads for each sample analyzed by RNA-sequencing*

**Sample**	**Medium**	**Reads**
Met_neg_1	BMM	803313
Met_neg_2	BMM	726389
Met_neg_3	BMM	596414
Met_pos_1	BMM + Methionine	696835
Met_pos_2	BMM + Methionine	536542
Met_pos_3	BMM + Methionine	756652
